# Effect of Innovative Food Processing Technologies on the Physicochemical and Nutritional Properties and Quality of Non-Dairy Plant-Based Beverages

**DOI:** 10.3390/foods9030288

**Published:** 2020-03-04

**Authors:** Paulo E. S. Munekata, Rubén Domínguez, Sravanthi Budaraju, Elena Roselló-Soto, Francisco J. Barba, Kumar Mallikarjunan, Shahin Roohinejad, José M. Lorenzo

**Affiliations:** 1 Centro Tecnológico de la Carne de Galicia, rúa Galicia n° 4, Parque Tecnológico de Galicia, San Cibrao das Viñas, 32900 Ourense, Spain; paulosichetti@ceteca.net (P.E.S.M.); rubendominguez@ceteca.net (R.D.); 2 Department of Food Science and Nutrition, University of Minnesota, St. Paul, MN 55108, USA; budar006@umn.edu (S.B.); kumarpm@umn.edu (K.M.); falcon.roh@gmail.com (S.R.); 3 Nutrition and Food Science Area, Preventive Medicine and Public Health, Food Science, Toxicology and Forensic Medicine Department, Faculty of Pharmacy, Universitat de València, Avda. Vicent Andrés Estellés, s/n 46100 Burjassot, València, Spainfrancisco.barba@uv.es (F.J.B.); 4 Burn and Wound Healing Research Center, Division of Food and Nutrition, Shiraz University of Medical Sciences, 71348-14336 Shiraz, Iran

**Keywords:** plant-based beverage, thermal treatments, non-thermal processing technologies, sensorial properties

## Abstract

Increase in allergenicity towards cow’s milk, lactose intolerance, the prevalence of hypercholesterolemia, and flexitarian choice of food consumption have increased the market for cow’s milk alternatives. Non-dairy plant-based beverages are useful alternatives because of the presence of bioactive components with health-promoting properties, which attract health-conscious consumers. However, the reduced nutritional value and sensory acceptability of the plant-based beverages (such as flavor, taste, and solubility) compared to cow’s milk pose a big threat to its place in the market. Thermal treatments are commonly used to ensure the quality of plant-based beverages during storage. However, the application of high temperatures can promote the degradation of thermolabile compounds and some detrimental reactions, thus reducing protein digestibility and amino acid availability of non-dairy plant-based beverages substitutes. New and advanced food processing technologies, such as high-pressure processing, high-pressure homogenization, pulsed electric fields, and ultrasound, are being researched for addressing the issues related to shelf life increase, emulsion stability, preservation of nutritional content and sensorial acceptability of the final product. However, the literature available on the application of non-thermal processing technologies on the physicochemical and nutritional properties of plant-based beverages is scarce. Concerted research efforts are required in the coming years in the functional plant-based beverages sector to prepare newer, tailor-made products which are palatable as well as nutritionally adequate.

## 1. Introduction

Over the last two decades, the consumption of non-dairy plant-based beverages, traditionally referred as “vegetable milks”, coming from legumes (soybeans), cereals (rice and oats) or nuts (almonds and hazelnuts), has increased considerably [1] due to health and environmental concerns, lactose intolerance, and flexitarian choice of food consumption, despite their taste [2]. According to Markets and Markets [1], the estimated growth of plant-based beverage alternatives is 15% by 2018, reaching a value of $14 billion. Comparatively, the market value of non-dairy plant-based beverages is expected to achieve around 5% of the market of milk and dairy products in the next 6 years [3,4]. Plant-based beverage alternatives are used not only as “milks”, but they are also extensively utilized in recipes as ingredients.

Typically, all the plant-based beverages are fluids that result from the process of maceration, grinding, and filtration, which consists of extracting food substances with water (accounting for about 90% of the final product). The traditional plant-based “milk” term is confusing from a nutritional point of view. Although the nutritional value of plant-based “milks” cannot be compared with cow’s milk, some of these non-dairy plant based beverages present great interest as a source of proteins, such as that observed for quinoa and soymilk (Table 1). Unlike cow’s milk, plant-based beverages contain no lactose or cholesterol, and they are usually sold with added calcium and vitamins, especially vitamin B_12_. The most common sources for plant-based beverages include cereal—(oat, rice, corn, spelt, rye, quinoa, oats, spelt, kamut), legume—(soy, peanut, lupin, cowpea), nut—(almond, coconut, hazelnut, pistachio, walnut), seed—(sesame, flax, sunflower, pumpkin, hemp), and pseudocereal-based (quinoa, teff, amaranth). Recent findings have drawn attention to the utilization of cereals, oilseeds, and nuts for new applications due to their functional properties (due to the large content of bioactive compounds present in these foods), which reveal the physical attributes of food components and their interactions [5].

**Figure 1:**
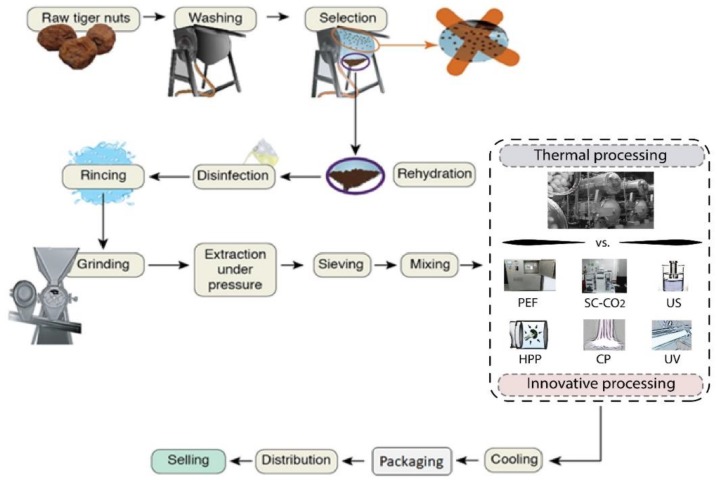
Innovative technologies with the potential to be used in the preservation of plant-based beverages (adapted from Roselló-Soto et al. [7]).

Plant-based beverages are usually subjected to ultra-high-temperature (UHT) treatment before being packaged to ensure food quality and safety during storage. However, the conventional high-temperature processing can promote the degradation of thermolabile compounds and some detrimental reactions, thus affecting the physicochemical properties and improving the off-flavor formation, for instance [6]. In order to reduce these drawbacks, alternative processing technologies such as high hydrostatic pressure, high-pressure homogenization, ultrasound, and pulsed electric fields have been proposed. The scientific evaluation of these innovative processing technologies revealed promising results to improve the shelf life, preserve the nutritional properties and reduce the loss of bioactive compounds of the plant-based beverages [5]. Some of the most relevant thermal and non-thermal innovative technologies which could be used for plant-based milk preservation as well as their potential applications are shown in Figure 1.

However, based on our knowledge, in comparison with dairy products, little scientific literature is available on the application of these technologies for plant-based beverages. Thus, this review outlines the intervention of various innovative processing technologies and their effect on the physicochemical and nutritional properties of plant-based beverages. The current limitations and suggestions for further studies regarding innovative processing technologies are also highlighted.

## 2. The Nutritional and Bioactive Composition of the Most Commonly Consumed Plant-Based Beverages

Plant-based beverages are quite variable in their composition compared to cow’s milk. The nutrients and amount of sugar in plant-based milk differ considerably according to the production process and product formulation. The nutritional composition of the most commonly consumed plant-based beverages and their characteristics are listed in Table 1. Among the different type of plant-based beverages, rice and sesame milks have the highest total carbohydrates (>15%) and calorie amount (>130 calories), while quinoa and soy milks contain the highest protein level (>4%).

Plant-based beverages are rich in bioactive compounds, but the particular bioactive compound differs according to the type of beverage. The bioactive compounds found in this type of products include: β-glucan, which is associated with improved blood glucose and insulin resistance [8]; phytosterols, which can improve the cardiovascular status and reduce the risk associated with related diseases [9]; isoflavones, which can reduce the risk related to development of cardiovascular diseases, cancer, and osteoporosis [10]; lignans, having the ability to reduce the blood cholesterol level [11]; and omega-3 fatty acids, which are involved in the adequate development and protection against the development of cardiovascular diseases, for instance [12]. Conversely, some antinutritional compounds can be also found in plant-based beverages. For instance, sesame and oat contain oxalate and phytates [8,11] that form insoluble complexes with minerals, carbohydrates and lipids during digestion and reduced availability of these nutrients [13]. Another important compound is amandin, an allergenic protein found in almonds [14].

**Figure 2:**
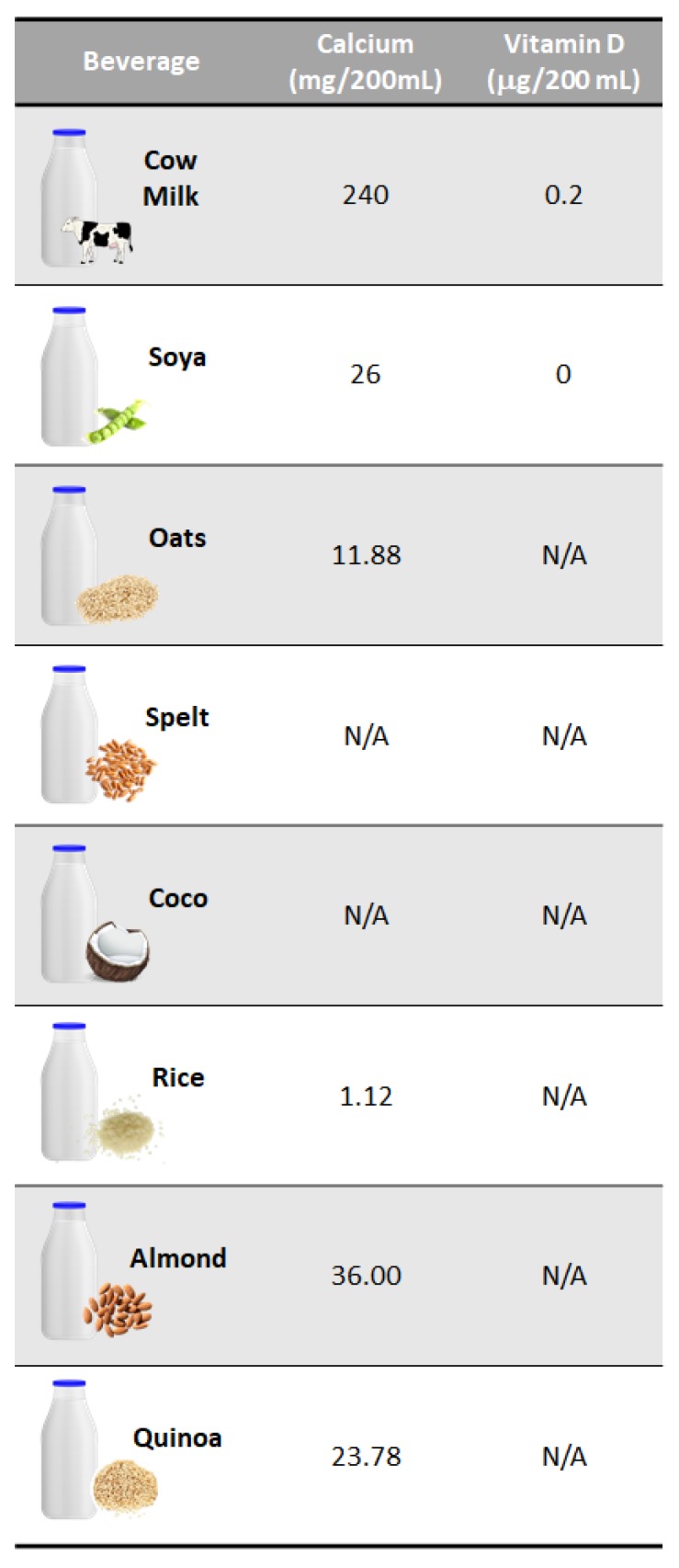
Calcium and vitamin D contents of non-dairy plant-based beverages compared to cow’s milk (values obtained from Sousa et al. [23]).

Moreover, from a nutritional point of view and taking into account the new trends found in some countries reducing the consumption of cow’s milk while increasing the consumption of non-dairy plant-based beverages, it should be emphasized that the nutritional value of plant-based beverages differ by far from that of cow’s milk [23]. For instance, it should be borne in mind that replacing cow’s milk with non-dairy plant-based beverages leads to a reduced intake of protein, calcium, certain vitamins (i.e., vitamin D) and minerals as well as a higher intake of added salt (Table 1 and Figure 2). In addition, from a physiological point of view, the intake of cow’s milk has nothing to do with plant-based beverages, due to the different nutritional composition. Therefore, taking into account the actual calcium, magnesium, and vitamin D deficits, such substitution should be done with caution and it is necessary to compensate for nutrient deficiencies in a balanced way using other food sources, in case it is necessary to replace cow’s milk, either for reasons of intolerance, allergies or others not related (e.g., religious, ethical, social).

## 3. Application of Various Innovative Technologies for the Processing of Plant-Based Beverages

### 3.1. High-Hydrostatic-Pressure Processing

The history of using high hydrostatic pressure (HHP) goes back to the 19th century when Soxhlet applied pressure to convert starch into glucose. HHP involves the use of pressure in the range of 100–800 MPa, with or without the application of heat [24,25]. In practical applications, the combined intensity of both thermal and pressure effects can cause various physical, chemical, and biological changes in foods. HHP can be conducted in either batch or semicontinuous process depending on the type of food. The use of HHP alters the characteristics of milks, particularly proteins, and leads to desired texture, sensory attributes, and nutritional value [26]. 

The effect of HHP can also contribute to reducing the allergenic character of some plant-based beverages. For instance, the impact of HHP on allergenic almond proteins was evaluated by Dhakal et al. [14]. In this study, the stability of the main almond allergenic protein amandin in the raw almond milk was evaluated under HHP treatment (450 and 600 MPa for 0, 30, 60, 180, 300, and 600 s at 30 °C) and traditional thermal processing (0, 30, 180, and 300 s at 72, 85, and 99 °C). The results showed that amandin levels were affected by all HHP treatments. The reactivity with antilinear epitopes of monoclonal antibodies was reduced by half while the signal from specific anticonformational monoclonal antibodies was no longer detected in HHP-treated samples. One of the explanations indicated by authors for these results was the aggregation of amandin, which can potentially reduce the immunological response in amandin-sensitive people. It is worth noting that similar results for conventional thermal processing were reported for treatments at increased temperature and time of 300 s at 85 and 99 °C. Additionally, the authors highlighted the necessity for further studies regarding the inactivation of allergenic proteins in almond milk, since HHP alone may not inactivate all allergenic factors. Combining HHP with other strategies such as changing pH, adding chemical additives, and increasing temperature could improve the inactivation of allergenic almond proteins, as stated by the authors.

The use of HHP also displayed an important role in the development of soymilk enriched with calcium [27]. In this experiment, a Doehlert design was used to optimize the HHP processing conditions (500–700 MPa at 73–95 °C) and level of calcium (5, 10, and 15 mmol/L) in order to enhance protein solubility and inactivation of trypsin inhibitors and lipoxygenase. The authors indicated that, in the optimum conditions (614 MPa, 85.5 °C, and 8.53 mmol Ca/L), both enzymes were completely inhibited and more than 70% of proteins were solubilized in the bulk of soymilk.

### 3.2. High-Pressure Homogenization

The high-pressure homogenization (HPH) or ultra-high-pressure homogenization (UHPH) is an alternative food processing method which can be used to improve the stability of plant-based milk emulsions and their physicochemical properties with minimum effect on the nutritional properties [22]. This technique uses high pressures ranging from 200 to 600 MPa and temperatures between 30 and 85 °C and can improve the stability of plant-based beverages by reducing the particle size of emulsions and producing more uniform size particles [28]. Apart from reducing colloidal particles, simultaneous destruction of microorganisms can be achieved using UHPH [29]. In a study performed by Briviba et al. [30], a threefold increase in mean particle size was reported, while no significant reduction in vitamin B_1_ and B_2_ contents after UHPH treatment (350 MPa at 85 °C) was observed. Moreover, the application of UHPH treatment resulted in reducing the almond protein antigens response by 99.8%.

The impact of UHPH (200–300 MPa) on the physicochemical properties (particle size and color) of soybean-based milks was compared with the results obtained from UHT-treated samples [29]. A significant reduction of particle size after UHPH was observed, but the aggregate formation was found only at 300 MPa. Although a partial protein denaturation after 200 MPa was observed, UHPH at 300 MPa led to similar protein denaturation to that found after UHT treatments. Moreover, UHPH soybean-based milks were more stable than UHT processed samples (particle aggregation during storage). Processing (UHPH and UHT), as well as storage time, had a significant influence on color parameters (CIE-Lab) wherein the lowest values were found in the samples treated by UHPH at 300 MPa (79.68, −2.66, and 16.01 for L, a*, and b*, respectively) in relation to UHPH at 200 MPa (82.24, −2.00, and 16.57 for L, a*, and b*, respectively) and UHT (84.31, −0.28, and 18.37 for L, a*, and b*, respectively).

In another study, the impact of UHPH (200–300 MPa/40–50 °C) on the properties of soybean-based milks was evaluated and compared with conventional thermal treatments (UHT and autoclaved) [31]. The authors evaluated the characteristics of soy-yogurts obtained from heat-treated soybean-based milks. The study showed an increased onset of gelation as well as decreased aggregation rate and gel network density on soybean-based milks treated by UHPH compared to heat treatments, which resulted in improved physiochemical properties (i.e., firmness).

Ferragut et al. [32] investigated the effect of UHPH treatment (200 MPa at 55 °C and 300 MPa at 75 °C) on the chemical composition, digestibility and essential amino acid content of an almond milk. The results of this study indicated non-significant differences in the chemical composition of almond milks subjected to both UHPH treatments and conventional thermal processing (pasteurization and ultra-high-temperature). It is worth mentioning that UHPH treatments did not affect the essential amino acid content, particularly lysine (a limiting amino acid in almond milks). However, the impact in a potential bioactive component can be greater than in chemical composition of plant-based beverages. In this sense, Toro-Funes et al. [33] evaluated the effect of UHPH on the stability of phytosterols, tocopherols, and polyamines in almond milk. UHPH (200 and 300 MPa at 55, 65, and 75 °C) treatment affected the content of phytosterols, tocopherols, and polyamines. Increasing pressure and temperature reduced the total tocopherol content (sum of α-, β-, and γ-tocopherol) from 50.63 to 3.15 and 4.14 mg/L, in raw almond milk treated at 200 MPa, 75 °C and 300 MPa, 75 °C, respectively. However, an inverse effect was reported for total phytosterols content, which increased from 22.08 mg/L in raw almond milk to 27.24 and 29.54 mg/L after UHPH treatment at 200 MPa, 75 °C and 300 MPa, 75 °C, respectively. According to the authors, this outcome could be derived from the mechanical forces (such as shear force, turbulence, and cavitation) reducing the fat globule size and facilitating the release of phytosterols in the bulk of the beverage.

Another important consideration is the impact of HPH on the volatile composition of plant-based beverages. The experiment carried out by Poliseli-Scopel et al. [34] evaluated the effect of UHPH (200 MPa, 55 or 75 °C and 300 MPa, 80 °C) on the volatile compounds profile of soybean-based milks and compared the results to those treated by the conventional pasteurization (90 °C, 30 s) and UHT (142 °C, 6 s). Apart from the type of treatment, hexanal was found to be the predominant compound [34]. Moreover, significant changes regarding the volatile compounds profile were observed after UHT and UHPH treatments wherein the highest modifications were observed after UHT treatments compared to untreated samples. Interestingly, Pérez-González et al. [35] used the headspace–solid-phase microextraction (HS-SPME) technique to characterize and detect changes in the volatile profile of almond milks arising from the technological treatments applied in the production of almond milk. The authors concluded that the predominant volatile compounds were benzaldehyde and hexanal, followed by ketone and alcohol. Interestingly, compounds such as furans and pyrazines (derived from thermal treatments) were detected to a lesser extent.

Likewise, some authors [36,37] compared the physicochemical and sensorial properties of UHPH-treated (200–300 MPa, 55–75 °C), pasteurized (90 °C, 30 s), and UHT (142 °C, 6 s) soymilk beverages. The application of UHPH at 200 MPa and 55 °C resulted in improved sensorial characteristics, color, and colloidal stability compared to samples treated with conventional thermal procedures [36,37]. Moreover, hydroperoxide index values were reduced and higher trypsin inhibitor activity in UHPH samples was observed compared to samples treated with conventional pasteurization and UHT techniques. A similar outcome was observed in a posteriori study carried out by Poliseli-Scopel et al. [37]. In the study, the impact of UHPH (300 MPa, 80 °C) and UHT (142 °C, 6 s) on soymilk beverage during storage were evaluated. The microbial growth was inhibited throughout storage time by both treatments, the hydroperoxide index was reduced during storage for both treatments, and sensory evaluation revealed slight differences between treatments that did not influence the panelists to differentiate treatments.

Toro-Funes et al. [38] evaluated the impact of UHPH (200–300 MPa, 55–75 °C) on the isoflavone profile, protein digestibility, and lysine availability immediately after the treatments and during subsequent refrigerated storage (4 °C) for 21 days. The results were compared to those obtained after conventional thermal pasteurization. The authors did not observe a significant modification in isoflavone profile after conventional pasteurization or UHPH processing compared to untreated samples. However, the conversion of isoflavone forms to aglycones during storage was reported, especially after conventional thermal pasteurization. Moreover, the percentage of blocked lysine was lower after UHPH, while no significant changes were found in protein digestibility during storage, independently of the applied treatment.

In a similar study, the same research group also compared the isoflavones and protein digestibility and lysine availability of UHPH (300 MPa and 75 °C of inlet temperature) and UHT-sterilized (142 °C, 6 s) soybean-based beverages, immediately after the processing and during 4 months of storage at 20 °C [39]. The isoflavones’ extractability was improved after UHT (≈38%) treatment compared to UHPH-treated samples (≈15%) immediately after processing. However, no significant differences were found in the total content of isoflavones at the end of the storage. Moreover, a faster interconversion of isoflavones into β-glucosides after UHT treatment was observed compared to UHPH-processed samples. A similar evolution of protein digestibility in both UHPH- and UHT-treated soybean-based beverages was found, being slightly higher in the initial UHT (88.4%) than in UHPH-treated samples (83.3%). Great differences were not observed in the percentage of blocked lysine among samples after treatments or in their evolution throughout storage [39]. Although important scientific advances were made in the characterization and storage stability of plant-based beverages processed by UHPH technology, further studies are necessary to optimize processing conditions to inactivate antinutritional factors and endogenous enzymes associated with quality decay.

### 3.3. Other Innovative Processing Technologies

The application of other advanced food treatments, processing parameters and the main effect on plant-based beverages are shown in detail in Table 2. Ultrasonication is another promising technology for homogenization of cow’s milk, but its application in plant-based beverages is poorly explored [40]. Iswarin and Permadi [41] investigated the effect of ultrasonic waves with different combinations of power levels (2.5 to 7.0 W) and exposure times (5 to 25 min) on droplet diameter of coconut-based milks. The diameter of the droplet size was reported to be reduced by increasing ultrasound’s power and time of processing. However, power levels were found to significantly affect particle size more than the duration of exposure.

The pulsed electric fields (PEF) is an interesting non-thermal technology used to inactivate microorganisms in different food products with minimum effect on flavor, color, or nutritional compounds [44]. Food is placed between two electrodes and subjected to very short pulses (for milliseconds/even in microseconds) at high voltages (1 to 80 kV/cm). Depending on the requirements (such as microbial inactivation, the disintegration of sludge, and electro-permeabilization), food products are subjected to various ranges of voltages [45]. PEF processing involves very short but strong pulses, thus shortening total processing time (TPT) with high efficiency. Common TPT is measured in minutes since pauses between pulses can be longer than pulse time itself [46]. 

Regarding the impact of such technology on plant-based beverages, Cortés et al. [42] investigated the effect of PEF processing on the quality attributes of “horchata” (tiger nut milk), a typical plant-based beverage from Spain [47]. The study evaluated the impact of treatment time (100–475 microseconds) and electric field intensity (20–35 kV/cm) during 5 days at 5 °C, while the temperatures during the treatment did not exceed 35 °C. According to the authors, the pH decrease during storage was ameliorated by PEF treatment that was even more influenced by treatment time. Samples subjected to longer PEF treatments, in the same electric field intensity, had higher pH values than samples subjected to short treatment times. The inhibition of peroxidase activity was another effect obtained from this experiment. Although the regeneration of such enzyme was observed in all PEF treatments, the enzymatic activity of peroxidase at the end of storage did not achieve the same level observed for untreated samples [42].

In the study conducted by Xiang [43], the rheological and color properties of the soybean-based milks were evaluated after PEF treatment wherein the effect of electric field intensity (18, 20, and 22 kV/cm) and number of pulses (25, 50, 75, and 100) at 26 °C were evaluated. The rheological properties of soybean-based milks were affected by PEF treatment. The apparent viscosity increased from 6.62 to 7.46 (10^−3^ Pa·s) by increasing the electric field intensity from 18 to 22 kV/cm and pulses from 0 to 100. The authors argued that this effect could be related to the formation of transient networks from the denatured molecules.

## 4. Effect of Innovative Processing Methods on Off-Flavor, Stability, and Shelf Life of Plant-Based Beverages

### 4.1. Removal and Prevention of Off-Flavor Generation

Among the non-dairy plant-based beverages, soy products are gaining importance due to their high-quality protein and positive effects on health. However, in order to increase the acceptability, several efforts have been made to reduce its displeasing, beany, or unpleasant flavor [48]. Unsaturated fatty acids and lipoxygenases are involved in the release of off-flavors in most of the plant-based beverages, which include soymilk [49]. In this sense, important strategies such as inactivating enzymes, removal of off-flavors by deodorization, and masking off-flavor by addition of artificial or natural flavorings are the most commonly used methods [5].

For instance, Zhang et al. [50] explored the effect of processing conditions and soy variety on selected volatile compounds of soymilk, particularly those derived from oxidative reactions. This study revealed that hot grinding (80.5 °C) combined with a two-phase UHT processing (vacuum evaporation at 50 kPa) was the most effective treatment to remove volatile compounds (such as hexanal, 2-pentylfuran, and (*E*,*E*)-2,4-nonadienal) from soymilk in comparison to low and ambient temperature grinding. The authors also argued that applying vacuum after thermal treatment (UHT process) of soymilk was considered as the main factor to achieve the low level of selected volatile compounds. Similarly, UHPH can effectively reduce the “beany” flavor in soymilk [34]. The authors observed that beany and grass sensory scores of UHPH-treated soymilks at 200 MPa/75 °C were lower than obtained by another processing condition (pasteurized and UHPH at 200 MPa/55 °C). Although many studies have shown promising approaches to eliminate “beany” and off-odor flavor from plant-based milks [51,52,53], more efforts are necessary to improve the actual frame and characterize the impact of new technologies and approaches.

### 4.2. Improving Product Stability

Product stability is an important parameter of plant-based milks. In general, during storage, the colloidal particles sediment or settle, making the plant-based milks unstable. Monitoring particle size can be of great value in order to understand the stability of plant-based beverages. The main consequence of particle aggregation and consequent precipitation is the impact on sensory properties of plant-based beverages, which can display lower scores regarding taste, consistency, and aroma [54]. Innovative food processing technologies display great potential to improve the storage stability of plant-based beverages. For instance, the use of UHPH technology (200 MPa at both 55 and 75 °C) prevented the sedimentation of suspended particles during refrigerated storage [34]. It is important to highlight that pasteurization improved the stabilization of suspended particles right after processing and during storage, but UHPH produced a more stable product. A similar outcome was reported for the processing of soymilk enriched with calcium treated with HHP [27]. In this case, the addition of calcium increased the sedimentation of particles after 5 days of storage, particularly for samples prepared with 15 mmol Ca/L and treated with conventional thermal processing (80 and 90 °C at 0.1 MPa). Conversely, the HHP samples (550 MPa at 83.7 °C and 650 MPa at 77.4 °C) displayed lower sedimentation after the same period in comparison to samples treated with conventional thermal processing and untreated soymilk. According to the authors, enhancing the solubilization of proteins and eventual formation of a protein network could explain the improved stability during storage.

Likewise, Bernat et al. [22] evaluated the impact of HPH and heat treatment (alone and combined treatments) on the particle size of almond and hazelnut milk. Increasing pressure on HPH treatment (up to 172 MPa) was associated with a lower volume mean diameter in comparison to untreated beverages than thermally treated beverages (85 °C for 30 min and at 121 °C for 15 min) for both beverages. The combination of HPH (172MPa at 85 °C for 30 min or 121 °C for 15 min) and thermal treatment partially reduced the volume mean diameter of a suspended particle of both beverages. Additionally, increasing temperature was associated with a higher volume mean diameter in the combined treatment, which according to authors supports the use of HPH followed by low thermal processing to improve the stability of almond and hazelnut beverages. It is worth mentioning that a study carried out by Cruz et al. [29] reported an interesting effect of HPH on the volume mean diameter of a suspended particle of soymilk. In this study, 200 MPa treatment reduced the volume mean diameter in comparison to untreated and UHT samples (0.13 vs. 0.55 and 0.47 µm, respectively) while the 300 MPa treatment induced a drastic increase in volume mean diameter (4.36 µm).

### 4.3. Shelf Life Improvement

Plant-based beverages are a rich source of nutrients for many microorganisms, which may affect product quality and safety during storage. The application of thermal treatments not only extends the shelf life of plant-based beverages but was also proven to improve taste and general acceptability [55]. One of the possible approaches to reduce the impact of heat treatment on the quality of plant-based beverages is combining effective time and temperature combinations. Several combinations have been tested so far: pasteurization at temperatures below 100 °C, sterilization at 121 °C for up to 20 min, and ultra-high temperature between 135 and 150 °C [5]. However, aseptic packaging is still necessary to preserve innocuity. Further storage at low temperature is required for pasteurized beverages, while sterilized products can be stored at room temperature [5,56].

The impact of innovative technologies on the shelf life of plant-based beverages is shown in Table 3. Several studies support the use of UHPH as a promising technology to improve the shelf life of plant-based beverages. The main advantage is the partial or integral inactivation of microbial load, which can increase the shelf life of plant-based beverages. For instance, UHPH (200 and 300 MPa, 40 °C) reduced the microbial load in tiger nut milk, particularly *Enterobacteriaceae*, *Lactobacillus*, molds, and yeasts [57]. The partial destruction of psychrotrophic and aerobic mesophilic bacteria was also reported by authors. However, no effect on mesophilic spores was observed on UHPH-treated tiger nut milk. Similar results for UHPH treatment were reported by other authors regarding the reduction of initial microbial load of freshly prepared plant-based beverages [28,29,34,36,37].

Similarly, Smith et al. [58] observed that HHP treatment reduced total bacterial count in a pressure-dependent manner wherein higher pressure yielded higher inhibition effect up to 4 days. After this period and up to 28 days of storage, total bacteria counts were similar among treatments. Differently, psychrotrophic bacteria were inactivated by HHP treatment at 75 °C with a pressure higher than 500 MPa, regardless of dwell time (1 or 5 min). Likewise, Poliseli-Scopel et al. [34] evaluated the effect of temperature (55 and 75 °C) during UHPH treatment (200 MPa) on the evolution of total bacteria and spores of soymilk. After 28 days of refrigerated storage, the soymilk treated at 75 °C displayed similar microbial load as observed after UHPH treatment (day 1) for both total bacteria and spores. A mild inhibition effect was observed for soymilk treated at 55 °C.

It is worth mentioning that other technologies can contribute to improving the safety of plant-based beverages such as PEF. For instance, *Escherichia coli* and *Staphylococcus aureus* loads in soymilk were reduced in a strength- and processing-time-dependent manner. The highest reduction (5.7 and 3.5 log_10_ reductions in *Escherichia coli* and *Staphylococcus aureus*, respectively) was obtained by treating inoculated soymilk with 40 kV for 547 µs [59]. Selma et al. [60] explored the impact of high-intensity PEF (2.5–3 MV/m, 50–300 µs, 5–16 °C) on horchata inoculated with *Enterobacter aerogenes*. The lag phase of treated horchata was improved in comparison to untreated samples. Particularly, for samples incubated at 16 °C, the lag phase obtained from untreated samples was 4.6 h, while in samples treated with high-intensity PEF, this lag phase was improved to 12.2–16.7 h. However, the authors obtained a maximum of 1.1 log reduction of *Enterobacter aerogenes* in treated samples. Likewise, Uemura et al. [61] inactivated *Bacillus subtilis* spores by applying radio-frequency flash heating treatment (a technology that heats food by electromagnetic radiation) in soymilk. The study revealed that by treating soymilk with 28 MHz, a reduction of 4 log in *Bacillus subtilis* spores was obtained. However, the feasibility of this technology, along with other non-thermal technologies such as pulsed light, to improve the shelf life of the plant-based beverages requires additional studies.

## 5. Challenges and Recommendations for Future Studies

Application of advanced food processing technologies like high pressure and PEF for the preservation of plant-based beverages presents numerous advantages over conventional heat treatment. However, the combination of innovative technologies with heating is still a major challenge for their successful consolidation in the plant-based beverage industry as processing technologies. In order to achieve this goal, more efforts are necessary to study the scale-up (since most of the technologies are currently at lab scale) and characterize the processing conditions at continuous regime with large flows, particularly for HPH technology.

Another relevant aspect that must be considered in further experiments is the effect of innovative technologies in the bioaccessibility of functional ingredients found or intentionally added in plant-based beverages for the development of new functional beverages. Scientific evidence indicated that phenolic compounds found in soymilk could be absorbed after the gastrointestinal digestion and exert antioxidant activity [62].

From this approach, the strategies for commercialization could be enhanced by viewing these products as a combination of benefits: primarily the individual relation to milk consumption (consumption of cholesterol- and/or lactose-free products, for instance) and the additional intention to health benefits (presence of bioactive compounds). This motivation mainly associated with young consumers, such as reported in the UK [63]. Therefore, both technological and consumer studies should consider targeting young consumers for the development of new products, the application of innovative technologies and characterization of this niche in the food market.

## 6. Conclusions

The plant-based beverages segment of food market has experienced enormous expansion prospective for the health food market and needs to be widely investigated through the development of advanced processing, technological interventions, and fortification techniques for developing nutritionally complete plant-based beverages with high overall acceptability. Plant-based beverages are associated with huge health and functional benefits, as discussed. Exploring advanced food processing technologies such as pulsed electric fields, high-pressure homogenization, and high-hydrostatic-pressure homogenization technologies can be helpful to tackle factors responsible for limiting the success in the processing such plant-based beverages on a larger scale. The effect of advanced technologies on plant-based beverages properties during both processing and storage require more studies in order to ensure that these technologies can prevent the loss of quality and promote the successful inactivation of antinutritional factors. Plant-based beverages will continue to be a major research area in the newer product development category of food science and technology.

## Figures and Tables

**Table 1:** Nutritional composition of most commonly consumed plant-based beverages per 8 oz cup along with their functional and antinutritional components.

Type of Milk	Calories (kcal)	Protein (g)	Lipids (g)	Total Carbohydrates (g)	Functional Compounds	Characteristics	Reference
Cow’s milk	61	3.15	3.25	4.8	-	-	[15]
Oat	80	2.5	4	-	β-Glucan	Antinutritional compounds such as phytates	[8,16,17]
Rice	130	1	2	27	Phytosterols (β-sitosterol and γ-oryzanol)	Poor emulsion stability due to high starch content	[9,18,19,20]
Quinoa	104	4.5	6	9	Manganese, Phosphorous	-	[9]
Soy	80	7	4	4	Isoflavones, Phytosterols	Beany flavor due to the action of lipoxygenase on unsaturated fatty acids	[10]
Almond	40	1	3	2	α-Tocopherol, Arabinose	Presence of allergenic protein amandin	[5,14]
Coconut	80	<1	5	7	Lauric acid, Vitamin E	-	[21]
Hazelnut	124	1.4	6	14	Catechin	-	[22]
Sesame	140	1.5	6	16.5	Lignans (sesamin, sesamolin, sesaminol)	Antinutritional factors such as oxalate	[11]
Hemp	70	2	6	1	Omega 3-fatty acids	-	[5]

**Table 2:** Application of innovative processing technologies and their effect on various plant-based beverages.

Novel Method	Plant-Based Milks	Treatment Conditions	Inference	Reference
High hydrostatic pressure (HHP)	Almond milk	HHP (450 and 600 MPa for 0, 30, 60, 180, 300, and 600 s at 30 °C) and traditional thermal processing (0, 30, 180, and 300 s at 72, 85, and 99 °C).	Induced the aggregation and coagulation of almond proteins; higher aggregation than conventional heat treatment (72 and 85 °C)	[26]
	Soymilk enriched with calcium	HHP (500–700 MPa at 73–95 °C) and traditional thermal processing (80, 85, 90, 95 °C at 0.1 MPa)	Inhibited the activity of trypsin inhibitors and lipoxygenase; improved protein solubility, viscosity, and stability (up to 5 days)	[27]
High-pressure (HPH)/Ultra-high-pressure homogenization (UHPH)	Almond milk	UHPH (350 MPa at 85 °C).	No significant reduction in vitamins B_1_ and B_2_. Reduction in anti-protein antigens by 99.8% was achieved	[30]
		UHPH (200 MPa at 55 °C and 300 MPa at 75 °C)	No significant difference in chemical composition, essential amino acid, lysine content (limiting amino acid in almond milk)	[32]
		UHPH (200 and 300 MPa at 55 °C, 65 °C and 75 °C)	Increased total phytosterols content; reduced the total tocopherol content	[38]
		HPH (150, 300, 450, and 600 MPa, 30 °C, up to 600 s) and traditional thermal processing (up to 300 s at 72, 85, and 99 °C)	Reduced protein solubility (up to 70%) and amandin immunoreactivity	[14]
	Soy yogurt	HPH (200–300 MPa at 40–50 °C)	Improved physiochemical properties (e.g., firmness) of the yogurts (milk treated by HPH)	[31]
	Soymilk	UHPH (200–300 MPa)	Improved stability during storage	[29]
		UHPH (200 MPa, 55 or 75 °C and 300 MPa, 80 °C)	Reduced hexanal formation using 200 MPa	[34]
		UHPH (200–300 MPa, 55–75 °C)	No effect on isoflavone profile after processing and protein digestibility during storage	[33,38]
		UHPH-treated soymilk (200 MPa, 55–75 °C) and thermal pasteurization (90 °C, 30 s)	Improved color and colloidal stability; reduced hydroperoxide index values and trypsin activity	[36,37]
		UHPH (300 MPa and 75 °C of inlet temperature) and UHT sterilization (142 °C, 6 s)	No significant difference in isoflavones extractability, protein digestibility	[39]
Ultrasonication (US)	Coconut milk	US power levels (2.5 to 7.0 W) and treatment time (5 to 25 min)	Droplet diameter was reduced by increasing US power and time	[41]
Pulsed Electric Field (PEF)	Tiger nut milk	PEF pulse time (100 µs and 475 µs) and electric field intensity (20 kV/cm and 35 kV/cm)	No change in fat content (3.04%) throughout the storage period; reduced formation of lipid oxidation products	[42]
	Soymilk	PEF with electric field intensities (18, 20, and 22 kV/cm), number of pulses (25, 50, 75, and 100), capacitance from the discharge capacitor of 0.33 L and pulse frequency of 0.5 Hz at 26 °C	Increased viscosity (22 kV/cm with 100 pulses)	[43]

**Table 3:** Effect of innovative processing technologies on shelf life of various plant-based beverages.

Novel Method	Plant-Based Milks	Treatment Conditions	Antimicrobial Effect	Reference
Ultrahigh pressure homogenization	Tiger nut milk	200 and 300 MPa at 40 °C	Total inhibition of *Enterobacteriaceae*, *Lactobacillus*, molds, and yeasts growth; partial inhibition of psychrotrophs and aerobic mesophiles growth, no effect on mesophilic spores	[57]
	Soymilk	200 and 300 MPa at 40 °C	Drastic reduction on total count, spores, and enterobacteria counts by both treatments	[29]
		200 and 300 MPa, 55–75 °C	Complete inhibition of total bacteria, total spores and *Bacillus cereus* growth by 200 MPa at 75 °C and 300 MPa at 55–75 °C	[36]
		200 MPa, 55 and 75 °C	200 MPa at 75 °C inhibited the growth of total bacteria and total spores during 28 days of refrigerated storage	[34]
		300 MPa at 80 °C	Complete inhibition of mesophilic and thermophilic bacteria	[37]
	Almond milk	200 and 300 MPa, 55–75 °C	Destruction of total bacteria, total spores, and *Bacillus cereus* growth by 200 MPa at 75 °C and 300 MPa at 55–75 °C	[28]
High-pressure processing	Soymilk	400–600 MPa, 25 and 75 °C, 1 and 5 min	Inhibition of total bacterial growth up to 4 days; 400–600 MPa at 75 °C caused total inhibition of aerobic bacteria for 28 days	[58]
Pulsed electric fields	Soymilk	20–40 kV/cm, 0–547 µs	Inactivation of *Escherichia coli* and *Staphylococcus aureus* was proportional to increasing strength and treatment time	[59]
High-intensity pulsed electric fields	Horchata	2.5–3 MV/m, 50–300 µs, 5–16 °C	Treatments increase the lag phase of *Enterobacter aerogenes* inoculated to horchata	[60]
Radio-frequency flash heating	Soymilk	3.5–28 MHz	4 log reduction in *Bacillus subtilis* spores by 28 MHz	[61]
